# Intraocular Inflammation Control and Changes in Retinal and Choroidal Architecture in Refractory Non-Infectious Uveitis Patients after Adalimumab Therapy

**DOI:** 10.3390/jcm9020510

**Published:** 2020-02-13

**Authors:** Vittorio Pirani, Paolo Pelliccioni, Serena De Turris, Alessandro Rosati, Alessandro Franceschi, Pierangelo Pasanisi, Rosaria Gesuita, Michele Nicolai, Cesare Mariotti

**Affiliations:** 1 Eye Clinic, Polytechnic University of Marche, via Conca 61, 60126 Ancona, Italy; piranivittorio@virgilio.it (V.P.); serena_deturris@hotmail.it (S.D.T.); alessandro.rosati8@gmail.com (A.R.); a.franceschi.md@gmail.com (A.F.); pierangelo.pasanisi@ospedaliriuniti.marche.it (P.P.); michele.nicolai@ospedaliriuniti.marche.it (M.N.); mariottiocul@gmail.com (C.M.); 2 Centre of Epidemiology and Biostatistics, Polytechnic University of Marche, via Conca 61, 60126 Ancona, Italy; r.gesuita@univpm.it

**Keywords:** Adalimumab, anti-TNF-α, non-infectious uveitis, refractory uveitis, inflammatory macular edema, macular thickness, choroidal thickness, retinal vasculitis, retinal hyperreflective spots

## Abstract

**Background**: Non-infectious uveitis represents a leading cause of visual impairment, and inflammation control represents a major priority in tackling visual acuity loss due to complications such as macular edema; different immunomodulatory drugs are currently being used, including anti-TNF-alpha Adalimumab. **Methods**: This was a monocentric observational study of 18 eyes of 18 patients with non-infectious uveitis treated with Adalimumab. The primary endpoint was the control of ocular inflammation. The secondary endpoints included the study of macular and choroidal thickness and architecture, visual acuity, changes in other treatments, and adverse effects. Results: Ocular inflammation was controlled at 12 months for 83.3% of patients. Central macular thickness improved from a median of 229.75 µm at baseline to 213 µm at 12 months, while choroidal thickness decreased by 11.54% at the end of the follow-up. A reduction of vasculitis on fluorescein angiography and of hyperreflective spots on optical coherence tomography was noted. Visual acuity also improved from 0.51 (logMAR) before treatment to 0.24 at more than 12 months (*p* = 0.01). A total of 11.1% of patients experienced side effects. **Conclusion**: Our study confirms the efficacy of adalimumab for the control of ocular inflammation, visual acuity preservation, and for corticosteroid sparing.

## 1. Introduction

Non-infectious uveitis (NIU) is reported to have affected more than 298.000 adults (estimated prevalence 121/100.000) and more than 21.000 children (estimated prevalence 29/100.000) in the United States in 2015 [1,2]. Audrey et al. [3] reported that NIU represents the fifth leading cause of visual impairment and it is accountable for about 10%–15% of legal blindness in middle-aged patients, predominantly between 20 and 50 years of age in developed countries [4,5,6] and up to 25% in the developing world [7,8,9].

Macular Edema (ME) represents the most common cause of sight loss among uveitis patients, because of its influence on “central vision” [7,10,11], and it has been found to be more common in those forms of uveitis that affect the more posterior structures of the eye [12]. Rothova et al. [13] showed that 45% of patients with posterior uveitis presented visual impairment, and 28% of them also had ME [4,13]. Uveitic ME is defined as a thickening of the macular region due to an inflammatory breakdown of the outer and/or inner blood–retina barrier (BRB) with consequent leaking from perifoveal capillaries and the accumulation of intracellular and extracellular fluid [14]. BRB breakdown may result from a strong inflammatory context and be due to many factors including vascular endothelium growth factor (VEGF), pro-inflammatory cytokines such as TNF-α, IL-1, TGF-beta, angiotensin II, IL-6, and IL-8, and metalloproteinases secreted by leukocytes [4,15,16].

NIU diagnosis is usually made based on clinical evaluation, blood testing, and multimodal imaging. Optical coherence tomography (OCT) has become the gold standard tool for the diagnosis of ME, since it is non-invasive, reproducible, rapid, and sensitive. It may accurately quantify macular and choroidal thickness and show fluid accumulation, the state of photoreceptor outer/inner segment line, hyperreflective dots, epiretinal membranes, and vitreomacular tractions [4]. Retinal fluorescein angiography (FA) is helpful for showing dye diffusion and dye pooling in the macular area, detecting ischaemic zones, and screening for associated vasculitis. Indocyanine green angiography (ICGA) is used to identify the inflammation of choroidal stroma and vessels [17,18]. A further diagnostic tool is fundus autofluorescence (FAF), which supplies insight into the metabolic state of the photoreceptors/retinal pigment epithelium complex, examining the presence of lipofuscin [17,19].

The treatment of NIU with inflammation control represents a major priority in tackling visual impairment due to complications such as ME [7,20], and it is the focus of our study. Corticosteroids have been traditionally used as first-line treatment [21] and are delivered by various routes, including systemic, local (periocular injections), and intraocular, with the limitation that they are associated with many side effects [22,23]. The second-line class of drugs includes non-corticosteroid immunomodulatory agents such as T-cell inhibitors (cyclosporine, tacrolimus), antimetabolites (azathioprine, mycophenolate mofetil, methotrexate), and alkylating agents (cyclophosphamide) [24,25,26,27]. In refractory cases, biologic agents such as interferons, immunoglobulins, and anti-Tumor Necrosis Factors have all been tried with varying degrees of success [28,29,30].

Adalimumab (Humira^®^; AbbVie Inc., North Chicago, IL, USA) received, in June 2016, Food and Drug Administration approval to treat adults with non-infectious intermediate, posterior, and panuveitis [3]. Adalimumab is a recombinant human IgG1 monoclonal antibody that targets human Tumor Necrosis Factor-α (TNF-α) [31]. The recent VISUAL trials [32,33,34] provided level 1 evidence supporting the clinical efficacy of this molecule in reducing the frequency of inflammation relapse for uveitis patients with a wide range of uveitic diagnoses [30,32,35].

In this study, we report our experience with the use of anti TNF-α (adalimumab) in patients affected by recalcitrant NIU by analyzing retinal and choroidal findings obtained by means of multimodal imaging after the administration of biologic systemic therapy.

## 2. Experimental Section

### 2.1. Study Population

This retrospective monocentric observational study was conducted at the Eye Clinic, Polytechnic University of Marche, Ancona, Italy. Thirty-five eyes of 18 patients with non-infectious uveitis treated with Adalimumab injections were enrolled from January 2017 to October 2019. The procedures of this study were in accordance with the Declaration of Helsinki and its later amendments. Written informed consent was obtained from all individual participants included in this paper, and the local Institutional Review Board (IRB) was informed (in accordance with the Italian Law in case of observational studies). All patients had uncontrolled uveitis at the time of the first anti-TNF-α administration. Uveitis was classified according to Standardization of Uveitis Nomenclature (SUN) criteria [36]: all cases presented chronic intermediate, posterior or panuveitis (Table 1). Most patients had been treated unsuccessfully with other immunosuppressive agents before the first Adalimumab injection.

### 2.2. Data Collection

The aim of the study was to evaluate the control of ocular inflammation and to observe choroidal and retinal changes after anti-TNFα injection. Anterior chamber cells, anterior chamber flare, and vitreous haze were evaluated and graded according to SUN criteria [36]. Parameters acquired included demographic information (age, gender), medical history, duration of follow-up, uveitis aetiology, previous and concomitant immunosuppressive drugs, date of the first injection, and adverse events. All the parameters were collected at baseline, after 3 months (3M), 6 months (6M), 9 months (9M), 12 months (12M), and, in some cases, over 12 months (>12M).

### 2.3. Adalimumab Administration

A complete physical evaluation and laboratory tests (complete blood cell count, serum protein electrophoresis, transaminases, hepatitis serology, C-reactive protein (CRP), QuantiFERON-TB-Gold^®^, antinuclear antibodies) and chest X-rays were performed before the beginning of Anti-TNF therapy to exclude hepatic toxicity or the presence of an infectious uveitis aetiology. Serological tests were repeated every three months in order to prevent Adalimumab’s adverse events. Adalimumab was administered as one subcutaneous injection (40 mg) every 2 weeks after an initial dose of 80 mg.

### 2.4. Ophthalmic Examination

All patients underwent a complete slit lamp examination with evaluation of the fundus, LogMar (Logarithm of the Minimum Angle of Resolution) Best Corrected Visual Acuity (BCVA) assessment and Intra-Ocular Pressure (IOP) measurements with applanation tonometry. OCT scans were collected with the Heidelberg Spectralis system (Heidelberg Engineering, Heidelberg, Germany) using the “Follow-up” function. This function allows the automatic detection of previous scans location, which are then used by the software to make subsequent image acquisitions. Macular and choroidal subfoveal thickness (micron) measurements were collected using the same scan at all follow-up visits. Fluorescein angiography (Heidelberg Retina Angiograph 2 (HRA2); Heidelberg Engineering, Heidelberg, Germany) and Indocyanine Green Angiography were performed in order to assess the presence of vasculitis, inflammatory choroidal neovascularization, or papilledema at baseline and at 12M and during the follow-up in accordance with an ophthalmological examination.

### 2.5. Statistical Analysis

Statistical analysis was performed using Statistical Package for Social Sciences (SPSS, version 22.0, IBM, Armonk, NY, USA). One eye for each patient was randomly chosen using the “Select Cases” function on SPSS. Qualitative variables were expressed as frequencies and percentages; quantitative variables were expressed using mean and standard deviation (SD). Median and IQR (interquartile ranges) were used to describe vasculitis (number of quadrants) and macular and choroidal thickness (micron). The Friedman test was used for multiple dependent comparisons with the post-hoc Wilcoxon signed rank test; Bonferroni adjustment was applied. A *p*-value ≤ 0.01 was considered statistically significant.

### 2.6. Efficacy Objectives

The primary objective was the control of ocular inflammation, defined by a lack of anterior chamber and vitreous cells, adhering to the SUN criteria, and a reduction of macular edema. The secondary objectives included the full analysis of the evolution of the mean macular thickness (MMT) and subfoveal choroidal thickness (SCT) in the central 1 mm area, measured using optical coherence tomography (OCT) (HRA 2-KT, Heidelberg Engineering, Heidelberg, Germany), the presence of vasculitis, neovascularizations or papilledema confirmed by indocyanine green angiography and fluorescein angiography (HRA 2, Heidelberg Engineering, Heidelberg, Germany). Moreover, changes in visual acuity and changes in other systemic or local treatments, including corticosteroid withdrawal, and modifications of retinal architecture were described.

## 3. Results

A total of 18 patients (18 eyes) were analysed. The mean age was 39 ± 13.40 years; 14 patients (78%) were males and four patients (22%) were females. The medium follow-up period was 22 ± 8 months (Minimum 12; Maximum 34).

Uveitis was bilateral in 99.44% of cases. Two patients had uveitis secondary to psoriatic arthritis (11.11%); 10 patients were affected by Behçet disease (55%) and three of them showed an overlapping syndrome (30%); three patients presented with pars planitis (16.66%); three patients had other forms of ocular inflammation (Table 1).

### 3.1. Inflammation Control

Fifteen patients (83.3%) presented successful inflammation control; at the final follow-up, there was lack of inflammatory activity according to SUN criteria (Table 2) or a reduction of inflammatory activity according to SUN criteria and reduction of the inflammation at the posterior pole (macular edema and vasculitis reduction). In fact, seven patients (38.9%) had a reduction in anterior chamber flare, eight patients (44.4%) of anterior chamber cells, and eight (44.4%) of vitreous haze.

Three patients (16.7%) did not reach the primary endpoint: two patients had side effects which led to therapy discontinuation (one of them having worsening of vitreous haze according to SUN criteria) and another patient did not show complete absence of intraocular inflammation. Despite a reduction in vitreous haze grade, according to SUN criteria, macular edema was still present.

In accordance with SUN definitions [36], inactive uveitis was achieved in 14 patients, and an improvement in uveitis could be seen in one additional patient.

### 3.2. Changes in Macular Thickness and Visual Acuity

A progressive decrease in macular thickness was observed after the initiation of the anti-TNF-α therapy from a median of 229.75 μm at M0 to 212.75 μm at M3, 209.5 μm at M6, and 213 μm at M12, i.e., a 7.29% reduction in the sample and 15.47% in the contralateral eye (Table 3). Furthermore, a significant increase in the mean best corrected visual acuity (log MAR) was observed after initiation of the anti-TNF-α therapy, with an improvement from 0.51 ± 0.6 at M0 to 0.24 ± 0.5 at M > 12 (Table 4), with a *p*-value of 0.01.

### 3.3. Changes in Choroidal Thickness

**Figure 1:**
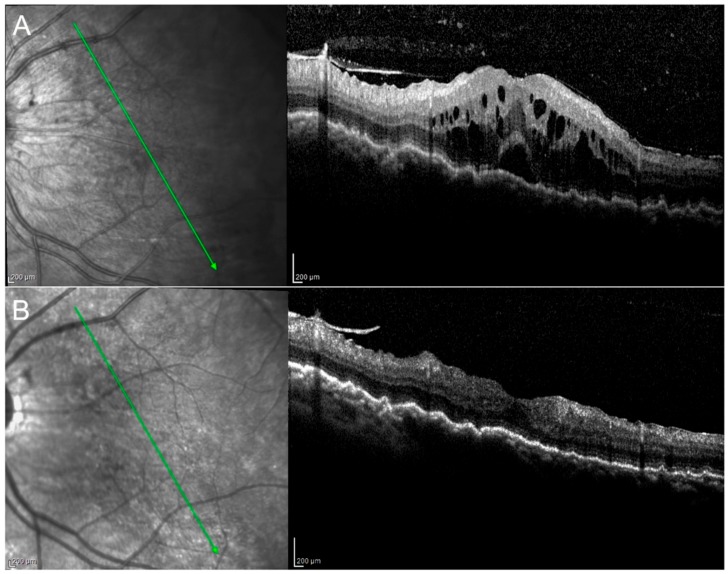
Birdshot retinopathy. Picture at baseline (**A**) shows the presence of macular edema and choroidal folds, which decreased at M3 (**B**). Vitritis (**A**) resolved at M3 (**B**).

A progressive decrease in choroidal thickness was observed after the initiation of the anti-TNF-α therapy from a median of 236.0 μm at M0 to 223.5 μm at M3, 208.75 μm at M6, and 208.75 μm at M12, i.e., an 11.54% reduction (Table 3). Choroidal folds could be seen in one patient at baseline and significantly reduced at M3 (Figure 1).

**Figure 2:**
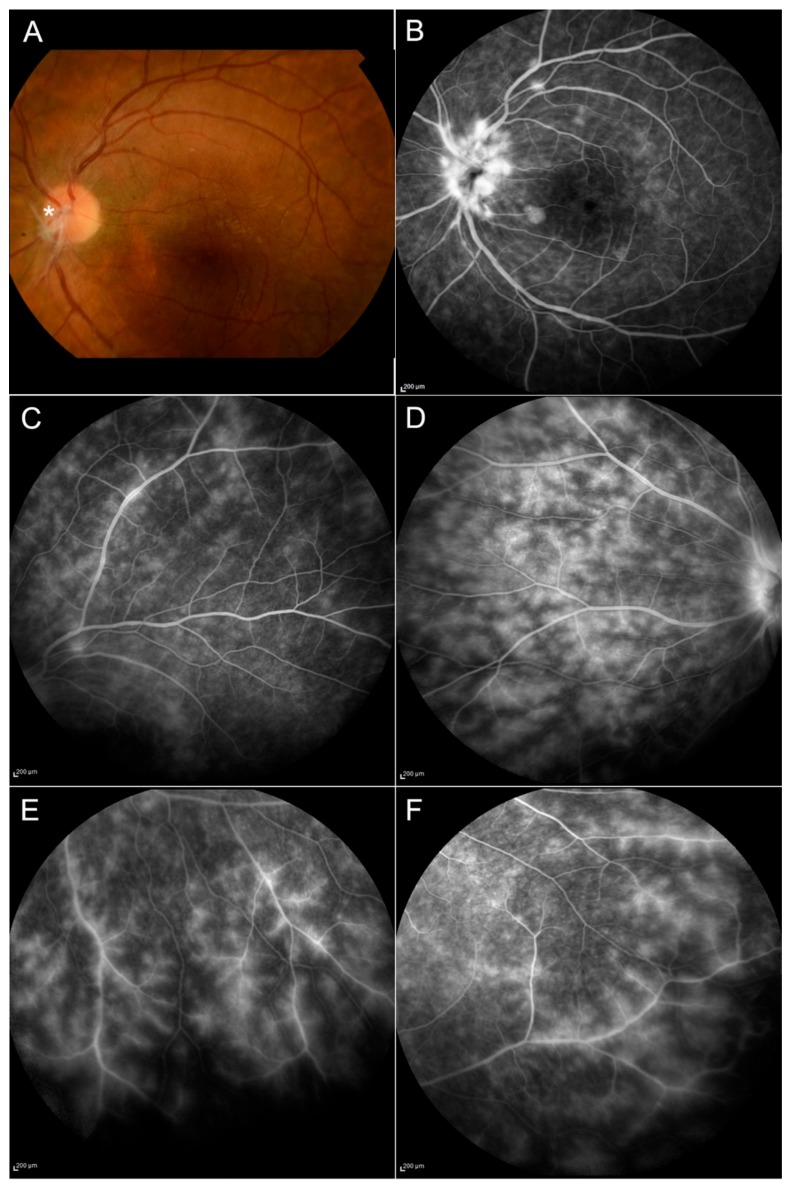
Behçet disease. Signs of active vasculitis can be seen in the posterior pole (**A**,**B**) and in the peripheral retina (**C**–**F**) with the presence of ischemic areas; papillitis is shown in (**B**). The fundus image displays the presence of hyalinized retinal vessels (**asterisk**) and an epiretinal membrane on the macula (**A**).

**Figure 3:**
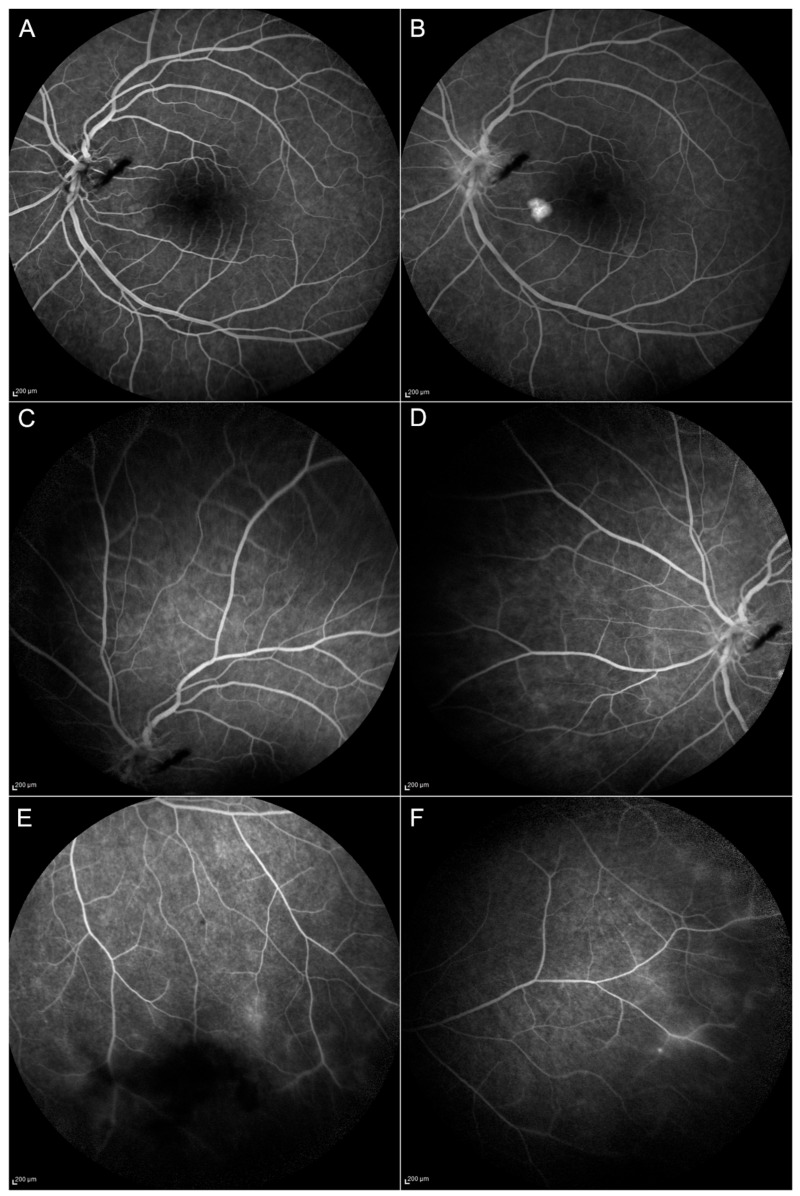
Follow-up at month 12 (M12) of the patient shown in Figure 2. Blood–retinal barrier alterations are still evident at the posterior pole from the early (**A**) to the late phases (**B**) of Fluorescein Angiography (FA). Peripheral vasculitis (**C**–**F**) and optic disc inflammation (**A**,**B**) are reduced compared to baseline but can still be appreciated in the inferior and the temporal sector of the peripheral retina (**E** and **F**, respectively).

**Figure 4:**
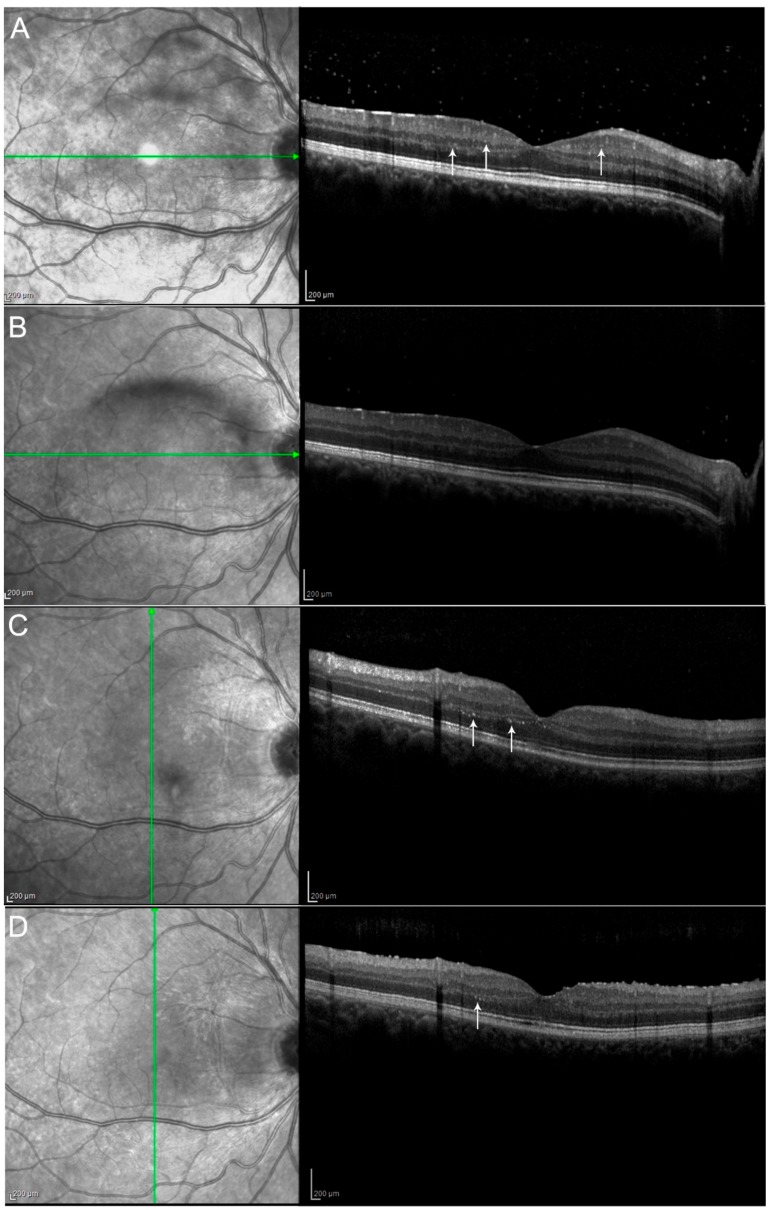
Hyperreflective retinal spots (see **arrows**) and vitritis shown at baseline (**A**) decreased one month after the beginning of the therapy with Adalimumab (**B**–**D**) show the same patient at M3 and M6 respectively with further improvement, although without complete disappearance of the spots. Hyperreflective spots have already been described as aggregates of activated microglial cells and could be considered a marker of neuroinflammatory response in the retina. They may also reflect retinal microvascular changes that occur in uveitic inflammation.

Results of the ophthalmic examination, OCT, and FA (presence of epiretinal membrane, retinal hyperreflective spots, papillitis, vitritis) are summarized in Table 4, and some selected examples can be seen in Figure 2, Figure 3 and Figure 4.

Granulomas were described at M0 in the patient affected by sarcoidosis and disappeared at M3. There was a significant reduction of vasculitis (number of quadrants affected) from M0 to M > 12 with a *p*-value of 0.01 (Table 3; Figure 3).

### 3.4. Changes in Systemic and Local Therapies

Previous immunosuppressive treatments are summarized in Table 1. For most patients (83.3%, *n* = 15/18) at M12, the systemic immunosuppressive therapy was reduced or completely discontinued with anti-TNF-α therapy. A total withdrawal of the corticosteroid therapy was obtained in 38.8% of patients (*n* = 7/18), four patients still received a daily dosage of prednisone over 10 mg during anti-TNF-α therapy, and none had to increase the corticosteroid therapy. Regarding other immunosuppressive treatments, nine patients had methotrexate, cyclosporin A or azathioprine associated with the anti-TNF-α therapy at M12 (Table 1).

### 3.5. Side Effects and Safety of the Anti-TNF-α Therapy

A total of 11.1% of patients (*n* = 2) experienced adverse events leading to treatment discontinuation: one patient reported diplopia which occurred after 14 months of anti-TNF-α therapy, while another had a worsening of vitreous inflammation and opacity after 15 months of treatment. Moreover, two patients complained of pain at the injection site with Adalimumab, while one patient reported erectile dysfunction.

## 4. Discussion

In this study, anti-TNF-α therapy with Adalimumab led to the effective and sustained control of ocular inflammation for 83.3% of patients at M12. According to SUN criteria anterior chamber flare, anterior chamber cells and vitreous haze grading decreased consistently.

Moreover, the anti-TNF-α therapy was effective in treating macular edema with a reduction of MMT at every follow-up visit. We observed a 7.29% reduction in MMT at M12 in the study eye and a 15.47% in the fellow eye. This is consistent with the key role of TNF-alpha in the regulation of ocular levels of different chemokines, including VEGF, TGF-beta, angiotensin II, IL-1, IL-6, and IL-8, which drive the development of macular edema and choroidal neovascularization. In parallel, there was a clear and statistically significant improvement in BCVA due to the reduction of macular thickness and vitritis. This is a very important result for patients, since improved visual acuity helps with the achievement of a better quality of life.

Another aspect which might have played a beneficial role in this choice was the fact that therapeutic adherence was reported as very high, as taking an incorrect dose or taking the medication at the wrong times was very difficult to achieve.

Another important parameter evaluated in this study is the change in SCT after treatment: in fact, a significant reduction was found, with a median value that reached 208.75 μm at M > 12 from baseline values of 236 μm. As choroidal circulation derives from systemic circulation, the eye might become a window through which to look at the whole organism, and to evaluate vascular modifications associated with systemic diseases [37].

During the active phase of the disease, the inflammatory vasodilation, first described by the Roman Cornelius Celsus, leads to an increase in choroidal thickness; this phenomenon, associated with an alteration in the outer blood–retinal barrier, may also explain the increase in macular thickness, due to a macula hyperhydration, also in absence of an evident macular edema [38].

Moreover, retinal circulation (which shares an embryological origin with cerebral circulation) must also be evaluated in these patients; in case of vascular retinal abnormalities, a thorough examination of the central nervous system must be performed with contrast-enhanced magnetic resonance imaging.

On fluorescein angiography, features of papillitis were found in four eyes at M0 and two eyes of one patient at the last follow-up visit, due to a chronic alteration of the outer blood–retina barrier.

There was a significant reduction in vasculitis over the four retinal quadrants from M0 to M>12 with a *p*-value of 0.01 and between the baseline and the other follow-up periods (Table 3).

Using optical coherence tomography, an inflammatory epiretinal membrane could be assessed in four eyes (22.22%) at M0; therefore, the observed decrease in MMT, caused by anti-TNF-α therapy with Adalimumab, might have been underestimated because of macular traction.

Neuroinflammation in the retinal layers has already been described in different diseases such as diabetes, retinal vein occlusion, and age-related macular degeneration [39,40,41]; these hyperreflective spots were interpreted as a microglial response to inflammation and to date, they have not been previously reported in these uveitic conditions and might be considered a sign of disease activity (Figure 3).

The withdrawal of systemic corticosteroids represents another critical need, because even though the control of the ocular inflammation can be obtained with low doses of prednisone, adverse side effects persist in the long term. In our study, a complete corticosteroid withdrawal with anti-TNF-α therapy was obtained in 38.8% of patients.

Anti-Adalimumab antibody formation with subsequent reduction of the response in patients treated with Adalimumab has been reported [42]; therefore, the addition of another immunomodulatory drug to anti-TNF monotherapy can reduce antibody formation and produce a synergic effect with Adalimumab, blocking different inflammatory pathways, even at a suboptimal dosage [43,44].

Moreover, in our Behçet patients, we chose not to add cyclosporine as an adjunctive immunomodulatory therapy as it seems to be associated with an increased risk of neurological involvement [45].

Anti-TNF-α may potentially induce severe side effects, including viral and bacterial infections, severe anaphylactic reactions, demyelinating neurological disorders, and the development of tumors (especially lymphoma).

In our study, 11.1% of patients (n = 2) experienced adverse events leading to treatment discontinuation; similarly, Adalimumab therapy was stopped in 9.5% of patients (n = 2) in the study by Mercier AE et al. [3].

Moreover, this might be considered a good rate when compared with other studies evaluating other anti-TNF-α drugs in the treatment of uveitis (19.3% of treatment discontinuation in the study performed by Kruh et al., and 19.4% in the study performed by Suhler et al.) [46,47].

In conclusion, the main limitations of this study are its retrospective nature and the fact that it is a single center study with a limited number of patients; however, our results showed the long-term effective control of Adalimumab in patients with noninfective uveitis, with preservation or even improvement in visual acuity.

Moreover, we report Adalimumab efficacy in reducing the hyperreflective retinal dots on optical coherence tomography (interpreted as a microglial response to inflammation, a sign of disease activity which, to date, has not been previously reported in these uveitic conditions).

An additional benefit was achieved in terms of ocular control and reduction of steroid dose and immunosuppressant drugs. Moreover, our data might help in the daily management of patients with noninfectious uveitis in a real-world setting.

## 5. Conclusions

Our study confirms the efficacy and safety of Adalimumab for the control of ocular inflammation in non-infectious uveitis and in particular when the uveitis is refractory to other therapy, including corticosteroids and immunosuppressive drugs. Moreover, additional data were given by means of multimodal imaging and may represent an opportunity for further research in this field. Visual acuity preservation and corticosteroids sparing represent additional benefits in the long-term management of these patients. For these reasons, it would be interesting to further investigate the efficacy of this anti-TNF-α therapy in large cohorts of patients affected by refractory non-infectious uveitis.

## Figures and Tables

**Table 1:** Clinical data of enrolled patients.

Patient No.	Etiology	Classification	Uni/Bilateral	Previous Treatment	Current Treatments	
					Drugs in Addition to Anti-TNF	Daily Steroid Dose > 10 mg
1	Psoriatic arthritis	Posterior	Bilateral	Etanercept	No	no
2	Psoriatic arthritis	Panuveitis	Bilateral	Prednisone, MTX	MTX	no
3	Behçet	Posterior	Unilateral	Prednisone, AZA	Prednisone	no
4	Behçet	Posterior	Bilateral	Prednisone, AZA	Prednisone	yes
5	Behçet	Posterior	Bilateral	Prednisone, AZA	Prednisone	yes
6	Behçet	Posterior	Bilateral	Prednisone, AZA	Prednisone	yes
7	Behçet	Panuveitis	Bilateral	Prednisone, AZA	AZA	no
8	Behçet	Panuveitis	Bilateral	Prednisone, MTX	Prednisone, MTX	yes
9	Behçet	Panuveitis	Bilateral	Prednisone, AZA	Prednisone	no
10	Behçet + Rheumatoid arthritis	Panuveitis	Bilateral	Prednisone, AZA	AZA	no
11	Behçet + Multifocal choroiditis	Posterior	Bilateral	Prednisone, AZA	AZA	no
12	Behçet, SLE	Posterior	Bilateral	AZA, Hydroxychloroquine	AZA	no
13	Birdshot Choroiditis	Panuveitis	Bilateral	Prednisone, AZA	AZA	no
14	Panuveitis	Panuveitis	Bilateral	Prednisone	Prednisone	no
15	Pars Planitis	Intermediate	Bilateral	Prednisone, CSA	CSA	no
16	Pars Planitis	Intermediate	Bilateral	Prednisone, CSA	Prednisone	no
17	Pars Planitis	Intermediate	Bilateral	Prednisone, AZA, CSA	CSA	no
18	Sarcoidosis	Panuveitis	Bilateral	Prednisone, AZA	Prednisone	no

AZA: Azathioprine; CSA: Cyclosporin A; MTX: Methotrexate.

**Table 2:** Patient evaluation according to SUN Criteria.

**Anterior Chamber Flare**		**1+**	**2+**	**3+**	**4+**	
**M0**	Patients (*n* = 8)	7	1	-	-	
**M12**	Patients (*n* = 1)	1	-	-	-	
**Anterior Chamber Cells**		**0.5+**	**1+**	**2+**	**3+**	**4+**
**M0**	Patients (*n* = 8)	3	4	1	-	-
**M12**	Patients (*n* = 1)	1	-	-	-	-
**Vitreous Haze**		**1**	**2**	**3**	**4**	**5**
**M0**	Patients (*n* = 9)	1	4	4	-	-
**M12**	Patients (*n* = 4)	3	-	1	-	-

**Table 3:** Macular thickness, choroidal thickness, and vasculitis at each evaluation.

	M0	M3	M6	M9	M12	M > 12
**Choroidal Thickness (micron)—Median**	236.0	223.5	208.75	223.5	208.75	208.75
IQR	260 302	263.0 338.75	257.5 305.75	251.5 296	236.5 286.25	238.5 288.5
*Friedman Test*	0.07					
*p*-value between baseline and last follow-up visit *(Wilcoxon signed-rank test)*	0.01					
**Macular Thickness (micron)—Median**	229.75	212.75	209.5	218.75	213	197.25
IQR	247 389	234.5 279	236 284.75	238 286.75	239.5 279.5	239.5 279.5
*Friedman Test*	0.35					
*p*-value between baseline and last follow-up visit *(Wilcoxon signed-rank test)*	0.07					
**Vasculitis (Number of Quadrants)—Median**	0	0	0	0	0	0
IQR	0 4	0 1.75	0 1	0 1	0 0.75	0 0
*Friedman Test*	<0.001					
*p*-value between baseline and other evaluations *(Wilcoxon signed-rank test with Bonferroni adjustment)*		0.04	**0.01**	**0.01**	**0.01**	**0.01**

**Table 4:** Sample features.

	M0	M > 12
**BCVA (LogMAR)**		
Mean	0.51	0.24
Standard Deviation	0.6	0.5
**ERM**		
Number	4	5
Percentage	22.22	27.78
**Spots**		
Number	4	2
Percentage	22.22	11.11
**Papillitis**		
Number	4	2
Percentage	22.22	11.11
**Vitritis**		
Number	9	4
Percentage	50.00	22.22

BCVA: Best Corrected Visual Acuity; LogMar: Logarithm of Minimum Angle of Resolution.
